# Emergence of Tuning to Natural Stimulus Statistics along the Central Auditory Pathway

**DOI:** 10.1371/journal.pone.0022584

**Published:** 2011-08-05

**Authors:** Jose A. Garcia-Lazaro, Bashir Ahmed, Jan W. H. Schnupp

**Affiliations:** Department of Physiology, Anatomy and Genetics, Sherrington Building, Parks Road, Oxford, United Kingdom; University of Southern California, United States of America

## Abstract

We have previously shown that neurons in primary auditory cortex (A1) of anaesthetized (ketamine/medetomidine) ferrets respond more strongly and reliably to dynamic stimuli whose statistics follow “natural” 1/*f* dynamics than to stimuli exhibiting pitch and amplitude modulations that are faster (1/*f* ^0.5^) or slower (1/*f* ^2^) than 1/*f.* To investigate where along the central auditory pathway this 1/*f*-modulation tuning arises, we have now characterized responses of neurons in the central nucleus of the inferior colliculus (ICC) and the ventral division of the mediate geniculate nucleus of the thalamus (MGV) to 1*/f* ^γ^ distributed stimuli with γ varying between 0.5 and 2.8. We found that, while the great majority of neurons recorded from the ICC showed a strong preference for the most rapidly varying (1/*f* ^0.5^ distributed) stimuli, responses from MGV neurons did not exhibit marked or systematic preferences for any particular γ exponent. Only in A1 did a majority of neurons respond with higher firing rates to stimuli in which γ takes values near 1. These results indicate that 1/*f* tuning emerges at forebrain levels of the ascending auditory pathway.

## Introduction

Natural sounds are complex and highly structured stimuli. A number of studies carried out on the auditory systems of insects [1], lower vertebrates [2] and mammals [3], [4], [5] have yielded evidence for evolutionary adaptations which exploit statistical properties of the natural acoustic environment in order to achieve efficient neural representations.

An interesting feature of natural sounds is that their second order statistics, i.e. fluctuations in intensity or pitch, are often characterized by 1*/f* spectra [3], [4], [6], [7], [8], [9], [10], where *f* is the frequency of the modulator. Voss and Clarke [8] presented random melodies with 1*/f* ^0^, 1*/f* and 1*/f* ^2^ pitch contours to several hundred listeners with varying levels of musicals skill and training, and found that listeners consistently preferred melodies with 1*/f* pitch contours over either 1*/f* ^0^ distributed ones, which were considered ‘too random’, and over 1*/f* ^2^ melodies, which were considered too slow or predictable.

1*/f* distributed signals are commonly found throughout the natural world, not just in the auditory modality. Yu et al. (2005) studied the responses of neurons in the visual cortex of macaque monkeys to sinusoidal gratings moving within the neurons' receptive fields with 1*/f* ^γ^ distributed random velocity profiles. Although their results suggest higher firing rates for 1*/f* ^0^ distributed, temporally uncorrelated signals, temporal transfer functions of V1 neurons exhibited higher gain, and the spike responses exhibited higher coding efficiency and information transmission rates for signals with *“*natural” 1*/f* temporal correlations than for 1*/f ^0^* or 1/*f* ^2^ (more strongly temporally correlated) signals.

Recently, we reported that neurons in primary auditory cortex (A1) respond most strongly and reliably if the statistics of the sound presented follows “natural” 1*/f* distributions [11]. These results raise the question whether this tuning is already found at lower stages of the auditory pathway. To investigate this, we recorded the responses of neurons in the ventral division of the medial geniculate nucleus (MGV) of the thalamus, and the central nucleus of the inferior colliculus (ICC) of anesthetized ferrets to synthetic stimuli featuring 1*/f* ^γ^ distributed frequency and amplitude modulations. The γ exponent determines the statistical dynamics of these stimuli, and was allowed to vary from 0.5 (very rapid fluctuations) to 2.8 (slow fluctuations). Using these *1/f* ^γ^ dynamic tone complexes, we characterized the responses of 379 units from the left ICC of three adult ferrets and 149 units from the left MGV of three further adult ferrets, and compare these data with 434 units recorded from A1. Identical recording methods were used at all three levels of the ascending auditory pathway. We observed marked differences in the responses elicited by our 1*/f* ^γ^ stimuli at these three levels of the central auditory pathway and conclude that a clear preference for values of γ only emerges at the level of cortex.

## Materials and Methods

### Ethics Statement

"The experimental protocols were reviewed by the Joint DPAG/Experimental Psychology Ethical review Committee and approved by the United Kingdom Home Office Inspectorate under project licence Nr 30/2664, in conformity with the 1986 Animals Scientific Procedures Act."

### Surgery and electrophysiological recording

Anaesthesia was induced by 2 ml/kg intramuscular injection of alphaxalone/alphadolone acetate (Saffan; Schering-Plough Animal Health, Welwyn Garden City, UK). The parietal and left temporal aspects of the skull were exposed, the skull was secured to a stainless steel head holder with stainless steel screws and dental acrylic, and a craniotomy was performed.

During electrophysiological recordings, anaesthesia was maintained with intravenous infusions of medetomidine (Domitor; Pfizer, Walton Oaks, Surrey)/ketamine (Ketaset; Fort Dodge Animal Health, Overland Park, Kansas, USA) at a typical rate of 0.022 and 5.0 mg/kg/hr respectively. (The anaesthetic dose was adjusted as required to maintain a stable level of anaesthesia.) Sterile 0.9% saline supplemented with 5% glucose was administered by i.v. infusion at a rate of 5 ml/h. The animals were artificially ventilated through a tracheal canula with oxygen-enriched air, and ECG, body core temperature and end-tidal CO_2_ were monitored throughout.

Recordings in the ICC were carried out using 2 MΩ 4×4 silicon array electrodes (Neuronexus Technologies, Ann Arbor, MI, USA) inserted after aspiration of overlying occipital cortex. Recordings in the ventral division of the medial geniculate nucleus (MGV) were carried out with the overlying cortex intact, using either 2 MΩ 4×4 or 2 MΩ 1×16 silicon array electrodes (Neuronexus Technologies, Ann Arbor, MI, USA) inserted 5 mm lateral to the midline and 2 mm posterior relative to the ear bar zero point (interaural axis).

Signals were digitized using TDT (Tucker Davis Technologies, Alachua, FL, USA) System 3 digital signal processors. BrainWare (TDT) was used to control stimulus presentation, data acquisition and to extract units of action potentials. Briefly, the shapes of each recorded action potential were automatically measured to determine a number of metrics (e.g. total amplitude of the action potential, the amplitude of the 1st or 2nd peak, the area under the spike, etc) that were plotted in a coordinate system. The axes of this system can be set to represent any of these metrics and clusters of dots representing the activity of single units can easily be identified. The basic concept in isolating single units is that action potentials from a single neuron have very similar shapes and cluster together provided the relative position of the neuron and the electrode remain constant, and that the neuron is not compromised in some way. The electrode signals were band-pass filtered (500 Hz –3 kHz), amplified (ca 15,000x) and digitized at 25 kHz. Data from responsive units were exported to Matlab (the MathWorks, Inc., Natick, MA, USA) for further analysis.

At the end of the recording experiments, the animals were overdosed with intravenous infusion of sodium pentobarbital (Euthatal, Merial Animal Health Ltd., Harlow, Essex, UK) and perfused through the heart with 4% paraformaldehyde in physiological saline to fix the neural tissues. The midbrain was removed from the skull, post-fixed and cryoprotected by immersion in a solution of 20% sucrose in saline for a minimum of 2 days. 50 µm slices were cut on a freezing microtome, mounted on microscope slides and Nissl stained using standard histological procedures. Electrode tracks were reconstructed from the Nissl stained sections to confirm that the electrodes had indeed been correctly placed in the ICC or MGV respectively.

The electrophysiological data from A1 described here had been recorded in the course of a previous study (Garcia-Lazaro et al., 2006) using identical recording procedures, and are reanalyzed here to facilitate the comparison across several stages of the auditory pathway.

### Acoustic stimuli

**Figure 1:**
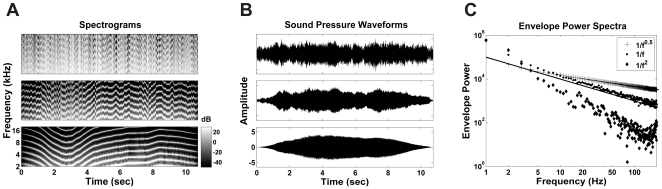
1/*f* ^γ^ stimuli. Spectrograms (A) and sound pressure waveforms (B) of three different 1/*f* ^γ^ tone complexes in which the values of γ were 0.5 (upper panel), 1 (middle panel) and 2 (lower panel). (C) Envelope power spectra for 1/*f* ^0.5^ (crosses), 1/*f* (circles) and 1/*f* ^2^ (diamonds) distributed stimuli. The solid line in the same plot illustrates an idealized 1/*f* spectrum.

The acoustic stimuli, (see [11] for a detailed description), consisted of randomly modulated tone complexes comprising tonal components spaced at third-octave intervals. We modulated both the frequencies (500 Hz to 20 kHz) and the intensities with statistically independent “random walk” profiles that were effectively “colored noise” generated using a standard inverse Fourier method. These “colored noise” or “random walk” modulators had amplitude spectra equal to *1/f* ^γ^ for *f* ≤94.5 Hz and zero for frequencies above 94.5 Hz, and phase spectra obtained from pseudo-random numbers drawn uniformly from the interval [0, 2π]. The 94.5 Hz “low-pass” on the modulation was introduced because amplitude modulators are well behaved only if their frequencies are significantly smaller than those of the lowest carrier (here 500 Hz). Using different random number seeds for the phase spectra, we were able to generate a variety of different stimuli for each exponent. In order to ensure statistical independence of the random series that determined the amplitude and the frequency modulation respectively, we also used different random seed values for each. The random walk series were 2^19^ points long. Given our chosen sample rate of 48828.125 Hz, this meant that our *1/f* ^γ^ stimuli were generated in 10.74 s long segments. The stimuli were delivered diotically through custom earphones (Panasonic RPHV297 drivers mounted on otoscopic speculae), using TDT System 3 digital signal processing equipment. Figure 1 illustrates spectrograms (A), sound pressure waveforms (B) and envelope power spectra (C) of three different sound stimuli where the values of the exponent γ were set to 0.5 (upper panels), 1 (middle panels) and 2 (lower panel). In (C), the traces representing 1/*f ^0.5^*, 1/*f ^1^* and 1/*f ^2^* dynamics are denoted by crosses, circles and diamonds respectively. The range of exponents tested was γ ∈ {0.5, 0.7, 1, 1.4, 2, 2.8}. Three different random walk stimuli were tested for each value of γ, and 5 responses were recorded for each stimulus. The 10.74 s long 1*/f* ^γ^ stimuli were presented at 15 s intervals (from onset to onset), allowing us to record 4.26 seconds of offset responses and spontaneous activity between subsequent stimulus presentations. Stimuli with different random number seeds and different exponents were randomly interleaved.

We used a simple Poisson regression model to test whether the units recorded were responsive to acoustic stimulation. This model treated stimulus as a ‘factor’ with 7 different ‘levels’ (i.e. the 6 different stimulus parameters plus the ‘null stimulus’ condition corresponding to the spontaneous activity). Spike counts during the first 4 seconds after stimulus onset for each of the 6 different stimulus types, as well as spontaneous spike counts were fitted with this Poisson regression model with 7 degrees of freedom (one for each stimulus type), as well as with a ‘null’ model which assumes no effect of stimulus condition on spike counts. An analysis of deviance was then used to decide whether the acoustic stimulation had a significant effect on spike counts, and only units which were shown in this manner to be responsive to the acoustic stimulation were included in the further analysis.

## Results

**Figure 2:**
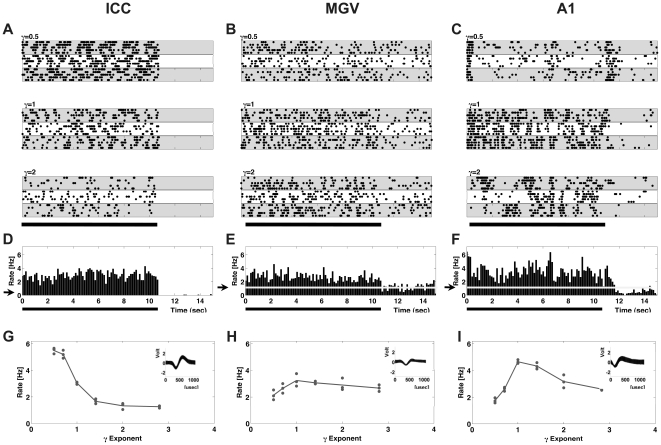
Responses from representative neurons. (A) Dot raster plots for the responses of one representative neuron recorded from the ICC. Each row of dots gives the times of action potentials to a single stimulus presentation. Each white and grey shaded band groups the responses to the five repeated presentations of each stimulus, so that each band shows the responses to a stimulus with a different random number seed. The black bar below the plots shows the duration of the stimuli. (B–C) Dot raster plots for the responses from representative neurons recorded from the MGV and A1 respectively. (D–F) Post-stimulus time histograms (PSTHs) for the same representative ICC, MGV and A1 neurons illustrated in (A–C). The PSTHs were pooled over all stimuli in the set. The level of the spontaneous firing rate in the PSTHs is indicated by the gray line and the black arrow on the ordinate. (G–I) Spike rate (Hz) as a function of the γ exponent for the same neurons illustrated in (A–C). Each data point represents the firing rate of the neuron averaged over five repeats to a different 1/*f* ^γ^ random walk stimulus. For each panel, the inset shows the action potential waveforms [potential (in units of volts) as a function of time (in µsec)] recorded from these neurons.

We characterized the responses of 379 units in the ICC, 149 units in the MGV and 434 units in the auditory cortex (A1) of 9 adult ferrets. Figure 2 shows dot raster plots for three representative units recorded from the ICC (A), MGV (B) and A1 (C) to stimuli in which the exponent γ took values of 0.5 (upper panels), 1 (middle panels) or 2 (lower panels). Each dot in the plot indicates the timing of an action potential relative to stimulus onset and each row of dots represents the response to a single stimulus presentation. The large majority of neurons in the ICC, like the example illustrated in Fig. 2A, exhibited higher sustained response rates for smaller values of γ. Spontaneous firing rates for ICC units were typically very low.

Response properties in the MGV were considerably more variable from one unit to the next, and spontaneous firing rates in the MGV were in most cases substantially higher than those seen in ICC. Fig. 2B shows the responses from one MGV unit, chosen to be ‘representative’, in as far as that is possible given the large response heterogeneity we observed in MGV. For stimuli with a value of γ = 0.5, this unit exhibited less sustained evoked activity than when the value of γ in the stimuli was set to 1.

In figure 2C, we show dot raster plots for responses from a representative cortical (A1) neuron for comparison. Clearly, for stimuli with γ = 0.5 (upper panel) this unit exhibits strong onset and offset responses, but adapts strongly and exhibits only a low sustained response rate. For γ = 1 (middle panel), responses are much more sustained, and offset responses are weaker. Like in the example of the thalamic unit illustrated in (B), it appears that this neuron exhibits less trial-to-trial variability in the responses for γ = 1 than for either γ = 0.5 or γ = 2.

Figure 2 (D–F) show the post-stimulus time histograms (PSTHs) for the same ICC, MGV and A1 units whose raster plots were shown in (A–C), where responses were averaged over all stimuli in the set. These PSTHs are typical examples in the following respects: Neurons at all three anatomical levels exhibited elevated firing rates throughout the duration of the stimuli (indicated by the dark line under the plot). Neurons in the ICC typically produced neither pronounced onset nor offset responses. This will be further illustrated below. A1 neurons, in contrast, showed marked onset responses, particularly for the more rapidly modulated stimuli with γ = 0.5. These onset responses were followed by sustained firing at a lower rate, as well as surprisingly vigorous and long lasting offset responses which could persist for approximately 1 sec after the stimulus ended. Such vigorous and prolonged offset responses were commonly observed in cortical neurons, but never in the ICC or MGV.

Figure 2 (G–I) plot the spike rate (Hz), averaged over the duration of the stimulus (from 0 to 10.74 s), against the γ exponent for the same units. Each dot represents the average response over the five repeats of one random walk stimulus. As was already apparent in the raster plots shown in Fig. 2A, the ICC unit shown in Fig. 2G responded with substantially higher firing rates to the more rapidly fluctuating stimuli with small values of γ. Figure 2H shows spike rates as a function of γ for the MGV unit illustrated in panels B & E. This unit exhibited the highest firing rates when γ  =  1, but the firing rate depended only weakly on γ, with similar firing rates observed for all values of γ tested. Figure 2I shows the γ-tuning curve for the cortical unit illustrated in panels C & F. This neuron exhibits clear evidence of tuning, responding with substantially higher firing rates to stimuli with values of γ close to 1.

**Figure 3:**
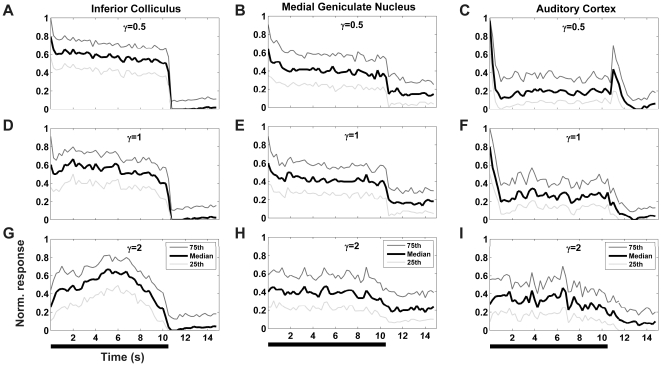
Population PSTHs. Normalized PSTHs, averaged over the population of midbrain, thalamic and cortical neurons, for stimuli with γ set to 0.5 (upper row), 1 (middle row) or 2 (lower row). The black middle trace in each panel represents the median response, the lower (light gray) and upper traces (dark gray) represent the 25^th^ and 75^th^ percentiles respectively. The thick black lines underneath the plots indicate the periods during which the stimuli were presented.

Figure 3 shows normalized PSTHs (relative to each unit's maximum evoked response rate), averaged over the entire population of units recorded at each of the three anatomical levels when γ was 0.5 (upper row), 1 (middle row) and 2 (lower row). In each panel, the middle trace shows the median, while the lower (in light gray) and upper traces (in dark gray) show the 25^th^ and 75^th^ percentiles respectively.

The PSTHs of the ICC neurons (figures 3A & D) exhibited small onset peaks for values of γ≤1. Neurons fired vigorously throughout the presentation of the stimulus and typically exhibited very low spontaneous firing rates. No onset response peaks were observed for γ = 2 (Fig. 3G), and no offset response peaks were observed in the ICC for any γ exponent tested.

Figure 3B, E & H show the normalized PSTHs averaged over the population of thalamic responses for γ = 0.5, 1 and 2 respectively. Again, small onset response peaks were observed for values of γ equal to or less than 1, but offset responses were not observed for any of the stimuli tested. Spontaneous firing rates for thalamic neurons were substantially higher than those observed for the ICC or A1.

Panels C, F and I in figure 3 show the normalized PSTH averaged over our sample population of A1 neurons. Cortical auditory neurons typically exhibited very marked onset response peaks but also fired vigorously throughout stimulus presentation. Offset responses were seen exclusively in A1 neurons, and were observed only when the value of γ in the stimulus took values less than one (Fig. 3C). In contrast, responses to stimuli that follow 1/*f* dynamics, (γ = 1, Fig. 3F), showed strong onset responses but no offset responses. Neither onset nor offset responses were observed when the value of γ was set to 2 (Fig. 3I).

**Figure 4:**
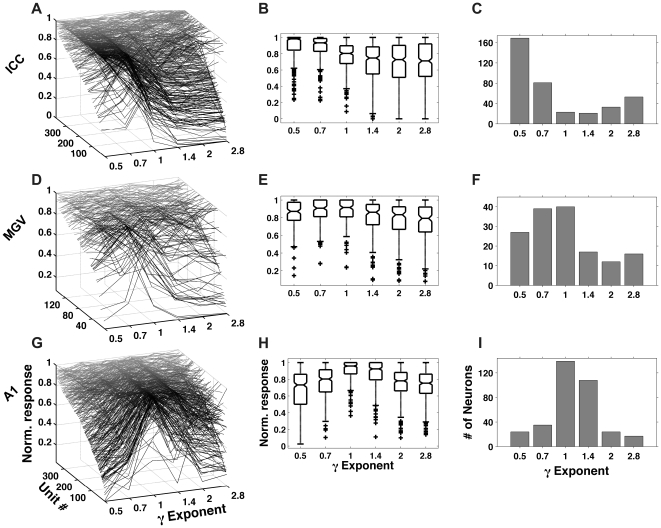
Population tuning curves. (A–C): Normalized γ tuning curves for our samples of ICC, MGV and A1 neurons respectively. Normalized response rates are plotted on the vertical (z-) axis (gray lines) and units were ranked and arranged along the depth (y-) axis according to γ tuning depth, i.e. tuning curves which exhibited large differences in their normalized firing rate as a function of γ are shown near the front and those with increasingly smaller differences in firing rates are plotted further back. (D–F): box-and-whisker plots showing the distribution of the normalized responses as a function of γ for the ICC, MGV and A1 neurons respectively. The lower and upper borders of each box denote the 25^th^ and 75^th^ percentiles of the sample and the line through the box indicates the sample median. The whiskers (lines at each end of the box) extend to show the most extreme data points that are not considered outliers (plotted individually using crosses). (G–I): Distribution of preferred values for γ ∈ {0.5, 0.7, 1, 1.4, 2, 2.8} over the sample populations of neurons recorded in the ICC, MGV and A1 respectively.

Figure 4 further summarizes and compares the neural responses observed at each of the anatomical stages we recorded from. Fig. 4A, shows γ-tuning curves for our entire sample population of ICC units as a 3D “waterfall plot”. Firing rates were averaged over the entire stimulus duration for each value of γ. The tuning curves were normalized relative to each unit's maximum evoked response rate, and plotted on the vertical (z-) axis (gray lines). Units were ranked and arranged along the depth (y-) axis according to “γ tuning depth”, i.e. tuning curves which exhibited large differences in their normalized firing rate as a function of γ are shown near the front and those with increasingly smaller dependence of firing rates on γ are plotted further back. The waterfall plot in Fig. 4A clearly shows that the large majority of ICC neurons responded most strongly to the smallest values of γ tested (0.5), although they could vary considerably in the depth of their tuning, and a minority of neurons preferred larger values of γ. The box and whisker plot in Fig. 4B shows the range of the distributions of the normalized ICC responses across the recorded sample population at the various values of γ tested. It confirms the observation that the large majority of ICC neurons responded preferentially to rapidly modulated stimuli, since the largest median normalized responses (> 0.9) were seen only for values of γ≤0.7. This dependence of response strength on the values of γ was statistically significant (ANOVA, *p*<10^−20^). The histogram in Fig. 4C shows the distribution of “preferred exponents” (those values of γ ∈ {0.5, 0.7, 1, 1.4, 2, 2.8} that evoked the strongest response) for the neurons recorded from the ICC. Clearly, the overwhelming majority of neurons responded most strongly when γ = 0.5.

In Fig. 4D we plot the normalized tuning curves for the sample population of neurons we recorded from the MGV. Unlike neurons in the ICC, MGV neurons do not exhibit strong trends, with many neurons showing flat tuning curves where the discernable peaks do not cluster very strongly around any particular value of γ. The box and whisker plot summarizing the distributions of the normalized responses for these data at each value of γ we tested is shown in Fig. 4E. This figure suggests a tendency for responses to be strongest on average for values of γ around 0.7–1, but the medians of the response distributions vary by less than 10% as a function of γ. Although these differences in response strength as a function γ are not as pronounced as those observed in the ICC, they are statistically significant (ANOVA, *p*<1.9867×10^−11^). The histogram in Fig. 4F shows the distribution of preferred exponents for MGV neurons. Unlike those in the ICC, preferences for any one value of γ are much less pronounced.

Figure 4G shows the γ tuning curves for the population of A1 neurons recorded from 2 animals in which the range of exponents tested was the same (γ ∈ {0.5, 0.7, 1, 1.4, 2, 2.8}). While there is variability across the population, particularly with respect to tuning depth, it is clear from Fig. 4G that the large majority of cortical neurons exhibit tuning to values of γ close to 1. In a further animal, the range of exponents we tested was narrowed to γ ∈ {0.5, 1, 2}. The data obtained from this animal exhibit similar trends and are shown in Figure S1. The box and whisker plot in Fig. 4H confirms that the largest median and 25^th^ percentile normalized responses for γ = 1. Again we observed very clear and statistically significant dependence of neural response strength on γ (ANOVA, *p*<2×10^−20^). Figure 4I shows the distribution of preferred exponents for the cortical neurons shown in Fig. 4G. The histogram shows that these neurons exhibit a clear preference for stimuli with “naturalistic” values of γ. Indeed, neurons preferring γ = 1 are at least 5 times more common than those with preferences for either γ = 0.5 or γ = 2.

The inset in figure 4I shows the histogram of preferred exponents for 75 neurons in which the range of exponents tested was γ ∈ {0.5, 1, 2}. Clearly, similar trends are observed where the number of neurons showing strong preference for γ = 1 are twice as common as those preferring γ = 0.5 or four times more common than those preferring γ = 2.

**Figure 5:**
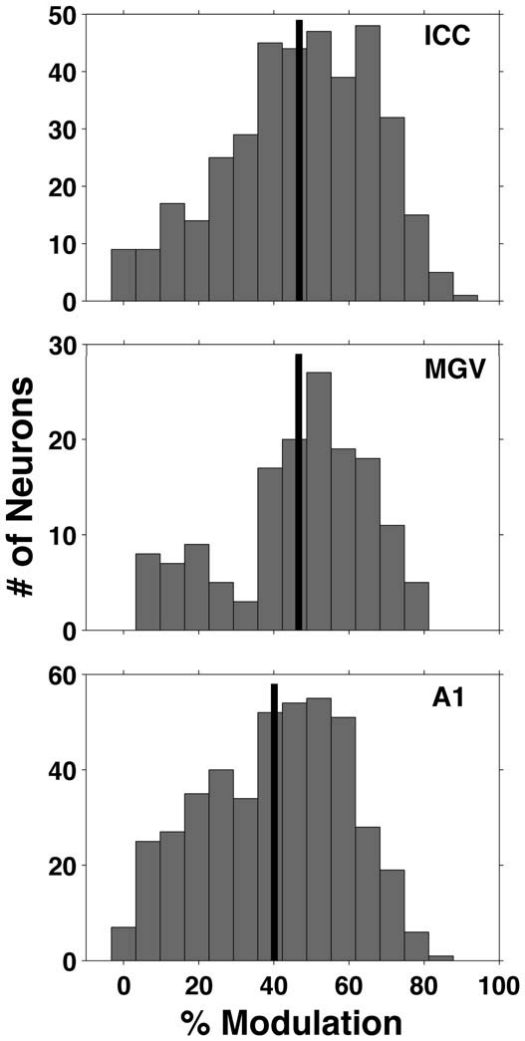
Response modulation. Histograms showing the modulation in the responses (calculated as described in the results section) exhibited by the entire sample population neurons recorded from the ICC (lower panel), MGV (middle panel) and A1 (upper panel) neurons respectively. The thick vertical black line indicates the mean values for each histogram.

To explore whether strongly γ tuned neurons might form distinct neural subpopulations, we examined the distribution of “γ tuning depths” exhibited by the population of neurons at the different anatomical levels we recorded from. The tuning depth was calculated as the ratio of the minimum to the maximum response for each neuron. The panels in Fig. 5 show histograms of the response modulations (calculated as a percentage) exhibited by neurons recorded from the ICC, MGV and A1 respectively. The observed distribution for the MGV shows a dip that is suggestive of bimodality, however, it is not statistically significant (Hartigan's dip test, *p* = 0.64). The tuning depth distributions at the three levels also exhibit clear similarities, as in the ICC, MGV and A1, tuning depths cover a wide range from 0 to 90%, and at all three anatomical levels, the tuning depth distributions peak between ca 40 and 70%. Thus, γ is clearly a similarly important stimulus parameter at levels of the ICC, MGV and A1, even if the predominant type of tuning to γ differs considerably and systematically at these three stations of the auditory pathway, favoring small γ at the level of ICC, and values of γ close to 1 at A1.

## Discussion

1/*f* signal statistics are a ubiquitous phenomenon observed in many physical systems. It has been observed, not just in natural “soundscapes” [7], [8], but also in many other physiological systems, including biomolecules [12], [13], ion channels [14], [15], cells of the heart [16], [17] and a diversity of neuronal [18], [19] and cognitive processes [20], [21].

Recently, we showed that neurons in the primary auditory cortex of the ferret [11] respond more strongly and reliably to dynamic stimuli whose statistics follow “natural” 1/*f* distributions. In this study we were able to show that this tuning to natural stimulus dynamics is not present in the major auditory relay nucleus of the midbrain, the ICC, but only emerges as auditory information travels through the MGV to primary auditory cortex. The large majority of ICC neurons responded most strongly to stimuli characterized by fast fluctuations in pitch and envelope, as exemplified by our stimuli with γ = 0.5. This result may seem surprising when considered in the context of other previously published work. Thus, Caspary et al., [22] reported that, when tested with sinusoidally amplitude modulated (SAM) tones, the majority of IC neurons exhibited low-pass modulation transfer functions, i.e. they generally responded more strongly at lower modulation frequencies. In contrast, we found that IC neurons on average clearly preferred our more rapidly modulated 1/*f* ^0.5^ random walk stimuli over the more slowly modulated ones. However, responses in IC to modulated stimuli are known to be highly complex, and depend on many variables, including modulation depth and overall sound intensity [23] which makes it very difficult to predict from rate modulation transfer functions recorded with CF SAM tones how these neurons would respond when tested with more complex, irregular and spectrally modulated stimuli such as ours.

Responses in the MGV, although they were often sensitive to changes in stimulus statistics, showed no consistent preference for any particular value of γ. A clear and pronounced preference for stimuli with 1/*f* statistics was only seen in primary auditory cortex, so this property is clearly not inherited from lower stations of the auditory pathway. Only a handful of studies so far (e.g. [24], [25], [26]) have directly compared the responses to the same stimulus sets under identical recording conditions both in the midbrain and cortex, and none of these studies would have led us to predict that tuning to naturalistic modulation spectra would emerge only at the level of the auditory cortex.

Some tuning to the statistics of the acoustic environment can be seen already at very early stages of the auditory pathway. For example, Lewicki's (2002) elegant analysis suggests that the auditory periphery may be set up so as to match the spectrotemporal statistics of our acoustic environment. Furthermore, in comparison to the visual system, many tuning properties are elaborated early in the ascending pathway. Thus, while for example binocular responses are visual receptive field properties which only emerge at the level of the primary visual cortex, binaural neurons are abundant in the auditory brainstem [27]. Our results identify the tuning to naturalistic temporal modulations as a rare example of a neural response property which arises *de novo* at the level of A1.

## Supporting Information

Figure S1 (A): 3D waterfall plot showing the normalized γ-tuning curves for 75 neurons recorded from one animal in which the range of exponents tested was γ ∈ {0.5, 1, 2}. Normalized response rates are plotted on the vertical (z-) axis (gray lines). Units were ranked and arranged along the depth (y-) axis according to γ-tuning depth. (B): Mean (± standard error) normalized response averaged over all neurons whose γ–tuning curves were shown in [A]). (C) Distribution of the exponents that evoked the strongest response for the same set of neurons. (D): Histogram showing the modulation in the responses (calculated as described in the results section) exhibited by the sample of neurons recorded from this animal. The thick vertical black line indicates the mean modulation value.
(TIF)
