# Supplementary figures and images for: Emergence of Tuning to Natural Stimulus Statistics along the Central Auditory Pathway
Source: PLoS One. 2011 Aug 5;6(8):e22584. doi: 10.1371/journal.pone.0022584 (PMC3151266; doi:10.1371/journal.pone.0022584)

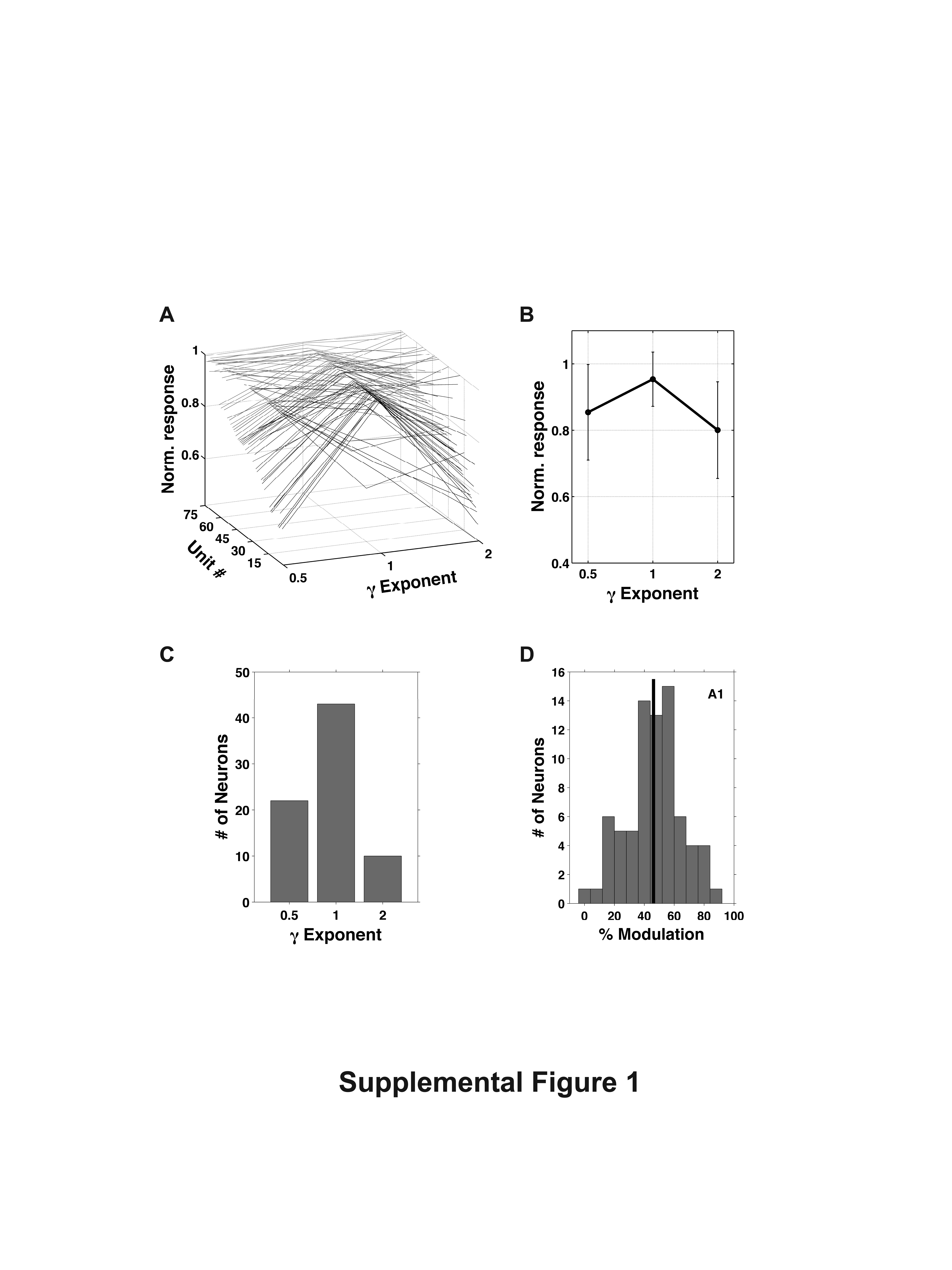

Supplement: Figure S1 — (A): 3D waterfall plot showing the normalized γ-tuning curves for 75 neurons recorded from one animal in which the range of exponents tested was γ ∈ {0.5, 1, 2}. Normalized response rates are plotted on the vertical (z-) axis (gray lines). Units were ranked and arranged along the depth (y-) axis according to γ-tuning depth. (B): Mean (± standard error) normalized response averaged over all neurons whose γ–tuning curves were shown in [A]). (C) Distribution of the exponents that evoked the strongest response for the same set of neurons. (D): Histogram showing the modulation in the responses (calculated as described in the results section) exhibited by the sample of neurons recorded from this animal. The thick vertical black line indicates the mean modulation value. (TIF) [file pone.0022584.s001.tif]
